# Prevalence of hallux valgus in the general population: a systematic review and meta-analysis

**DOI:** 10.1186/1757-1146-3-21

**Published:** 2010-09-27

**Authors:** Sheree Nix, Michelle Smith, Bill Vicenzino

**Affiliations:** 1 Division of Physiotherapy, School of Health and Rehabilitation Sciences, The University of Queensland, Brisbane, Australia

## Abstract

**Background:**

Hallux valgus (HV) is a foot deformity commonly seen in medical practice, often accompanied by significant functional disability and foot pain. Despite frequent mention in a diverse body of literature, a precise estimate of the prevalence of HV is difficult to ascertain. The purpose of this systematic review was to investigate prevalence of HV in the overall population and evaluate the influence of age and gender.

**Methods:**

Electronic databases (Medline, Embase, and CINAHL) and reference lists of included papers were searched to June 2009 for papers on HV prevalence without language restriction. MeSH terms and keywords were used relating to HV or bunions, prevalence and various synonyms. Included studies were surveys reporting original data for prevalence of HV or bunions in healthy populations of any age group. Surveys reporting prevalence data grouped with other foot deformities and in specific disease groups (e.g. rheumatoid arthritis, diabetes) were excluded. Two independent investigators quality rated all included papers on the Epidemiological Appraisal Instrument. Data on raw prevalence, population studied and methodology were extracted. Prevalence proportions and the standard error were calculated, and meta-analysis was performed using a random effects model.

**Results:**

A total of 78 papers reporting results of 76 surveys (total 496,957 participants) were included and grouped by study population for meta-analysis. Pooled prevalence estimates for HV were 23% in adults aged 18-65 years (CI: 16.3 to 29.6) and 35.7% in elderly people aged over 65 years (CI: 29.5 to 42.0). Prevalence increased with age and was higher in females [30% (CI: 22 to 38)] compared to males [13% (CI: 9 to 17)]. Potential sources of bias were sampling method, study quality and method of HV diagnosis.

**Conclusions:**

Notwithstanding the wide variation in estimates, it is evident that HV is prevalent; more so in females and with increasing age. Methodological quality issues need to be addressed in interpreting reports in the literature and in future research.

## Background

Hallux valgus (HV) is one of the most common chronic foot complaints presenting to foot and ankle specialists [1], occurring when the hallux deviates laterally towards the other toes, and the first metatarsal head becomes prominent medially [2]. As well as being a major contributor to the costs for forefoot surgery, HV has been linked to functional disability, including foot pain [3], impaired gait patterns [4], poor balance [5], and falls in older adults [6,7].

Although HV has gained substantial attention in both historic and recent literature, several authors have highlighted the fact that a true prevalence estimate for HV is difficult to ascertain [8,9]. A wide range of prevalence estimates for HV has been presented in a multitude of independent reports. National health surveys in the United States have reported a prevalence of 0.9% across all age groups [10], while a more recent survey in the UK reported a prevalence of 28.4% in adults [9]. Research conducted in elderly populations has indicated prevalence rates as high as 74% [11]. Individual studies have reported that HV is more common in female and elderly individuals [9,12]; however, there has been no synthesis of the literature to date or synopsis derived.

Due to the lack of firm epidemiological data relating to HV, it is difficult to estimate the impact that this condition has on the population; thus, in order to establish the need for future research, a better understanding of HV prevalence is warranted. To date there has been no published systematic review investigating the prevalence of HV and the influence of age and gender. Therefore, the aim of this systematic review and meta-analysis was to examine HV prevalence in the overall population and in age and gender subgroups.

## Methods

### Data sources

Electronic databases (Medline, Embase, and CINAHL) were searched by the first author for all years available up to June 2009 to identify all publications discussing HV prevalence. Broad MeSH terms and keywords were used combining the following: the condition of interest (e.g. *bunion *or *hallux valgus *or *great toe deformity *or *foot deformity *or *foot problem*) and epidemiological terms (e.g. *questionnaire *or *survey *or *prevalence *or *incidence)*. For the full search syntax with truncation used for each database refer to Additional file 1 (*Additional file 1.xls*). Reference lists of all included papers were hand-searched to identify grey literature (i.e. government publications and theses), articles that were too old to be indexed on electronic databases, and articles without abstracts that were missed by the initial search strategy.

### Study selection

**Figure 1:**
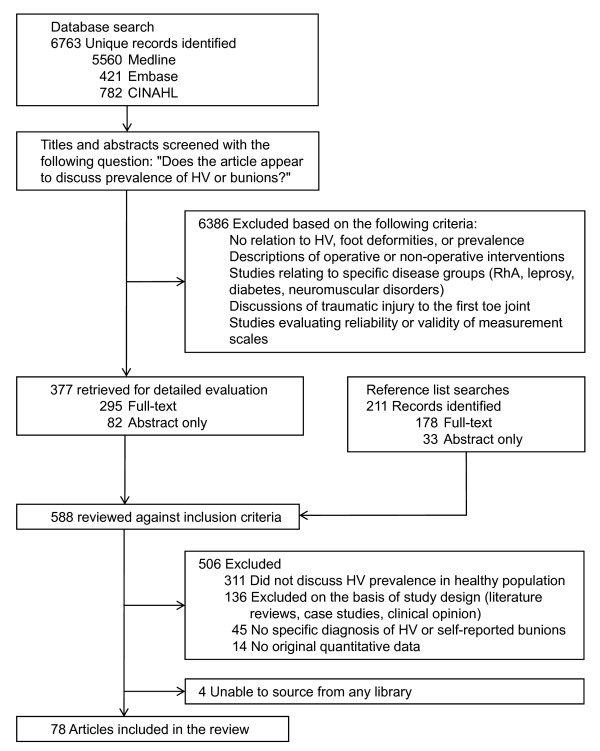
**Selection process for inclusion of articles in the review**.

All titles and abstracts retrieved by the above search strategy were scanned by the first author using an initial screening question: *Does the article appear to discuss prevalence of hallux valgus or bunions? *The full text was sourced if required, and the same author undertook detailed eligibility assessment using pre-determined criteria based on HV diagnosis (including both clinically diagnosed HV and self-reported bunions), study design, and reports of original quantitative data for HV prevalence (Figure 1). Surveys of specific disease groups (e.g. rheumatoid arthritis or diabetes), intervention studies, and studies where prevalence data was grouped with other foot deformities were excluded. As this review was not restricted to the English language, translations were sourced for articles written in German, Russian, Spanish, Serbian, Turkish, and Chinese.

### Quality assessment

Papers were scored for quality by two independent assessors using the Epidemiological Appraisal Instrument (EAI) [13], which has been shown to be a reliable and valid tool for assessing observational studies. Items not applicable to cross-sectional study designs were removed, resulting in a 17-item scale. Title, author and journal details were removed to de-identify articles prior to rating. Disagreements between the two assessors were resolved by consultation with a third party. Each item was scored as either "Yes" (score = 2), "Partial" (score = 1), "No" (score = 0), "Unable to determine" (score = 0), or "Not Applicable" (item was removed from scoring). Detailed criteria to determine each response were modified from the original instrument and agreed upon by all assessors prior to rating. The overall score was derived as an average of the scores for all 17 items (range 0-2). Studies were then classified as either "high" or "low" quality using the median quality score (0.91).

### Data extraction

Data extraction was performed by the first author, and queries discussed and resolved by all authors in regular meetings. Prevalence data were extracted for each study population and converted to raw counts of individuals with HV. Raw prevalence data for age and gender subgroups was also extracted separately wherever possible, as previously published literature has suggested that HV prevalence varies with these factors [9]. Authors were contacted where additional information was required.

### Statistical methods

The summary statistic for each study or subgroup was a prevalence proportion, calculated as the ratio of the number of individuals with HV to the sample size of that study or subgroup. The standard error for each prevalence estimate was then calculated. Meta-analysis was performed to obtain pooled prevalence estimates using a random effects model, which gives an average estimate across studies weighted by sample size. A Chi-squared test was used to determine heterogeneity across studies. Due to the diversity of study populations, prevalence estimates were only pooled between studies with similar age and gender characteristics. For the purposes of this age subgroup analysis, we categorised age by three broad categories: juvenile (< 18 years), adult (18-65 years), and elderly (> 65 years). Studies in which the sample did not exactly fall within one of these age categories were categorised independently by each author, and if a consensus could not be reached data were excluded from the age subgroup analysis.

The subgroup (24 studies) that reported HV prevalence for the overall population (i.e. all ages included in their sample, and a prevalence estimate given that was not split by gender or age) was further analysed for potential sources of bias. Studies were grouped according to sampling method, definition of HV, and study quality to determine if these factors influenced prevalence estimates. Influence of sample size and publication year were investigated by funnel plots. All analyses were performed using Stata version 10 [14].

## Results

### Database search

The database search yielded a total of 8456 hits, from which 1693 were removed as duplicates. The remaining 6763 citations were scanned by title and abstract, and 377 potentially relevant records were identified. Fifty-seven of these satisfied all eligibility criteria and gave original data for HV prevalence. Hand-searching of reference lists yielded another 211 potentially relevant titles, of which 21 met all eligibility criteria and were included in the review. A total of 78 papers were included and underwent quality assessment (Figure 1). Papers that reported on the same sample as a previously published study (n = 7) were only included once in the analysis. Four papers reported data from more than one sample population; thus, data were extracted from a total of 76 studies (total 496,957 participants). One author was contacted to provide clarification that multiple papers reported data from the same sample. Another author who only provided graphical data for age and gender subgroups was also contacted during data extraction.

### Study characteristics

Selected characteristics of all studies included in the review can be found in Additional file 2 (*Additional file 2.xls*). Study characteristics varied widely in terms of study population and methodology. Twenty-eight studies (37%) were conducted in the USA, 21 (28%) in the UK, 8 (10%) in Australia, and 4 (5%) in Germany, with the remaining 15 studies (20%) conducted in other regions. More than half of studies (66%) conducted a clinical examination, while others utilised interviews (13%) or questionnaires (7%) to gather self-report data. Fifteen studies (20%) were published after the year 2000, and 19 studies (25%) were published before 1970. Sample sizes varied widely, with the smallest sample reported being 30 individuals [15], and the largest sample being 197,422 individuals surveyed in a US National Health Survey [16].

### Quality assessment

Overall agreement for rating of quality of reporting and methodology between the two assessors was 87%. The results from the quality assessment can be found in Additional file 3 (*Additional file 3.xls*). The quality assessment revealed that only 18 studies (24%) used a random sampling method, only 39% of studies adequately described their sampling frame, and less than half of studies (47%) provided a simple description of study participant characteristics, such as age and gender. Despite the importance of a clear definition of HV, only twelve studies (16%) defined HV according to angular criteria. Reliability and validity of measurement methods were described in only five (7%) and four (5%) studies, respectively.

### Meta-analysis

**Figure 2:**
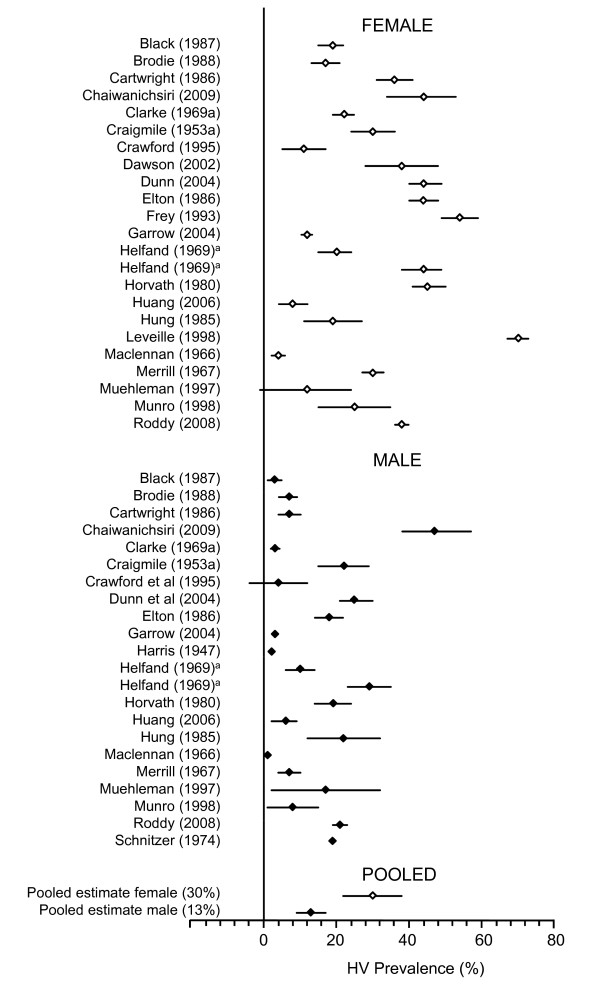
**HV prevalence estimates by gender**. Diamonds indicate prevalence estimates by male (black diamonds) and female (white diamonds) subgroups, with bars representing 95% confidence intervals. ^a ^Study reported more than one prevalence estimate based on different diagnostic methods in the same sample population (self-reported vs. clinically diagnosed HV).

Studies included in the meta-analysis, grouped by age of study population, are listed in Additional file 4 (*Additional file 4.xls*). Meta-analysis by age subgroups revealed a prevalence of 23% (CI: 16.3 to 29.6) in adults aged 18-65 years (15 studies), and HV prevalence clearly increased with age (Table 1). Studies that reported HV prevalence by gender consistently showed a higher prevalence of HV in females [30% (CI: 22 to 38)] (23 studies) compared to males [13% (CI: 9 to 17)] (22 studies) (Figure 2). However, there was a high degree of heterogeneity between studies in all subgroups (χ^2 ^156.55 to 3213.78; *p *< 0.0001; I^2 ^= 95.8% to 99.6%).

**Table 1:** Pooled random effects estimates for HV prevalence by age subgroup expressed as % (95% CI)

	**Overall**	**Male**	**Female**
	
**Juvenile**			
Pooled prevalence estimate	7.8 (6.2 to 9.5)	5.7 (3.7 to 7.6)	15.0 (7.7 to 22.3)
Number of studies	16	5	6
			
**Adult**			
Pooled prevalence estimate	23.0 (16.3 to 29.6)	8.5 (1.4 to 15.6)	26.3 (16.5 to 36.2)
Number of studies	15	8	9
			
**Elderly**			
Pooled prevalence estimate	35.7 (29.5 to 42.0)	16.0 (10.6 to 21.3)	36.0 (26.9 to 45.1)
Number of studies	37	16	16

**Figure 3:**
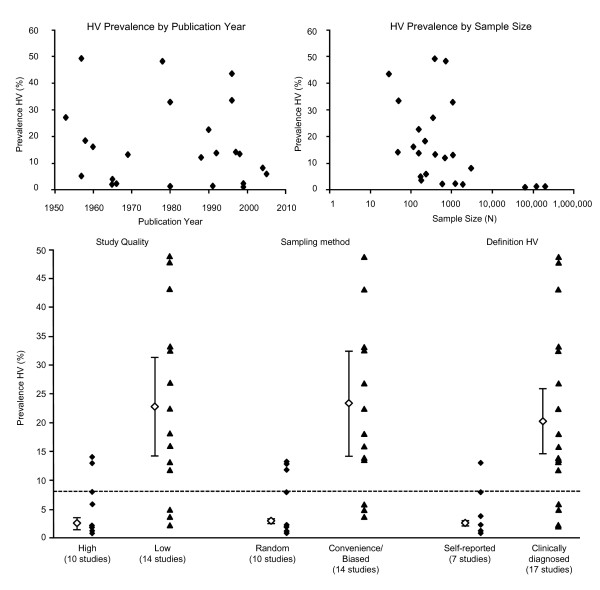
**Potential sources of bias in reported HV prevalence in the overall population (based on 24 studies)**. Clear diamonds indicate pooled random effects estimate by subgroup; error bars represent 95% confidence intervals; dotted line represents an overall pooled estimate, although there was significant heterogeneity across the 24 studies.

Finally, prevalence estimates were influenced by method of HV diagnosis (self-report or clinically diagnosed), sampling methods (random, convenience, or biased) and study quality. Studies using self-report data and random sampling methods, as well as those with high quality scores on the EAI reported lower prevalence estimates. There was no consistent trend apparent with regard to sample size or publication year (Figure 3).

## Discussion

This review revealed a wide variation in HV prevalence estimates, and meta-analysis showed that systematic differences in these estimates were related to a number of factors, including method of HV diagnosis, gender, age, study quality, and sampling method. The finding that substantial differences may be related to the method of HV diagnosis (i.e. self-report or clinical examination) (Figure 3), confirms the results of a number of studies that have shown lower prevalence rates with the self-report methods commonly used in large-scale surveys when directly compared to clinical examination [17-22]. Prevalence of HV may therefore be under-reported in epidemiological surveys that rely on self-report data.

Systematic differences according to gender and age were clearly demonstrated by our meta-analysis. The pooled estimate of HV prevalence in females (30%) was 2.3 times greater than the estimate for males (13%). This supports the observation of several individual reports that HV is more prevalent in females. For example, a recent large-scale epidemiological study of people older than 30 years reported a prevalence of 38% in women compared to 21% in men [9], and another recent survey of older adults reported a prevalence of 58% in women and 25% in men [12]. The trend for an increase in HV prevalence with age was also demonstrated by our data: 7.8% in juveniles (16 studies, n = 73,030), 23% in adults aged 18-65 years (15 studies, n = 23,790) and 35.7% in the elderly (37 studies, n = 16,001) (Table 1).

Variations in reported prevalence of HV in previous literature may also be explained by differences in study quality and methodological issues, particularly sampling bias (Figure 3). We identified a trend for higher prevalence estimates from studies with low quality scores on the EAI (score <0.91). Higher prevalence estimates were also reported by studies using convenience samples [23-29] or biased samples of people seeking treatment for foot problems [15,30,31], in comparison to those studies that used random sampling from the general population [10,16,32-39]. Potential bias may be introduced by lower quality studies with sampling bias; however, as discussed previously, this trend may also be related to the fact that these "low" quality studies were mostly clinical studies that diagnosed HV rather than relying on self-report data.

Our findings should be considered in light of several limitations in the available literature concerning HV. One major concern is the lack of a clearly stated definition of HV in the majority of studies reviewed. Even in those studies where HV was observed on clinical examination, very few described a quantifiable method of measuring HV. Only 16% of studies in our review defined a diagnosis of HV using angular criteria measured clinically or on x-ray. A few more recent studies used the Manchester Scale, a categorical scale based on standardised photographs with four gradings to classify HV severity [40-43]. Of those studies that collected self-reported prevalence data via interview or questionnaire, only a few provided participants with a definition or diagram of HV [9,35,44]. In addition, there is confusion surrounding the interchangeable use of the terms "bunion" and "hallux valgus." In this review both terms were considered to represent HV; however, the term "bunion" strictly refers to the medial bursitis that may develop over the first metatarsal head as a result of irritation [1]. Most included studies that used self-report data asked subjects about "bunions"; undoubtedly, a poor understanding of the terms used in a questionnaire or interview will result in inaccurate self-report data. Finally, there has been poor reporting of the reliability and validity of methods used to diagnose HV. Clearly, for accurate prevalence data to be collected and compared across different populations a consistent definition of HV and validated measurements should be employed.

Another consideration for our meta-analysis was the statistically significant degree of heterogeneity or variation across studies. Wide variations in sample populations meant that much of the retrieved data could not be pooled; however, pooling of estimates across age and gender subgroups was considered to be an important synopsis of the available literature pertaining to HV. Our subgroup meta-analysis was limited by the fact that not all studies reported HV prevalence by gender or age. Those studies that did report prevalence by age used a range of different age groupings, which rendered impossible further sub grouping the 18-65 years age bracket. Our analysis of potential sources of bias (Figure 3) was conducted to attempt to explain this variation between studies and highlight possible sources of heterogeneity.

Finally, insufficient data was available to examine the influence or adjust for other factors such as ethnicity, geographic location, shoe wearing or socioeconomic status on HV prevalence. Details of sampling frame and sample characteristics were also often poorly reported, as revealed by our quality assessment (Additional file 3 *Additional File 3.xls*). The vast majority of studies did not report on the presence of symptoms (i.e. pain or disability) related to HV, and therefore this factor could not be investigated by our review.

Having highlighted the limitations of the currently available epidemiological data relating to HV, further large-scale epidemiological studies are clearly warranted. Future studies should utilise rigorous methods, including random sampling from the general population and from different ethnic and socioeconomic groups. Validated tools should be used to diagnose HV, and results should be reported by gender and age as these factors are known to be associated with HV prevalence. Information relating to the presence of symptomatic versus asymptomatic HV would also be of great benefit in determining the impact of HV on the general population. Clear reporting of all these factors in future studies will provide an evidence base that will enhance our understanding of the impact of HV on the population and the health care system, and subsequently assist with the delivery of appropriate treatment. Due to its prevalence in the aging population, further research should focus on the impact of HV on mobility and quality of life in the elderly.

## Conclusions

This meta-analysis reveals a high prevalence of HV in the overall population and highlights the wide variation in prevalence estimates across studies. Our results also support the commonly held view that HV is more prevalent in women and the elderly. This study has highlighted the issues that make it difficult to provide a true estimate of HV prevalence in the general population, with recommendations for future research.

## Competing interests

The authors declare that they have no competing interests.

## Authors' contributions

All authors contributed equally to the conception and design of this study. SN carried out literature searches, quality assessments, data extraction and statistical analysis and was responsible for drafting of the manuscript. MS also carried out quality assessments. MS and BV were responsible for supervision, including interpretation of data and critical revision of the manuscript. All authors read and approved the final manuscript.

## Supplementary Material

Additional file 1 **Search syntax used for electronic databases**. Click here for file

Additional file 2 **Selected characteristics of papers included in systematic review **[4,5,9-11,15-40,42-87]. Click here for file

Additional file 3 **Results from quality assessment**. Click here for file

Additional file 4 **Studies included in meta-analysis, grouped by age of study population**. Click here for file
